# Emergence of a High-Risk *Klebsiella michiganensis* Clone Disseminating Carbapenemase Genes

**DOI:** 10.3389/fmicb.2022.880248

**Published:** 2022-05-23

**Authors:** Isaac Prah, Yoko Nukui, Shoji Yamaoka, Ryoichi Saito

**Affiliations:** ^1^Department of Molecular Microbiology, Graduate School of Medicine and Dental Science, Tokyo Medical and Dental University, Tokyo, Japan; ^2^Department of Molecular Virology, Graduate School of Medicine and Dental Science, Tokyo Medical and Dental University, Tokyo, Japan; ^3^Department of Infection Control and Prevention, Tokyo Medical and Dental University Hospital, Tokyo, Japan; ^4^Department of Infection Control and Laboratory Medicine, Kyoto Prefectural University of Medicine, Kyoto, Japan

**Keywords:** *Klebsiella michiganensis*, IncX3-*bla*_NDM-5_ plasmid, fitness cost, stability, KoI

## Abstract

*Klebsiella michiganensis* is emerging as an important human pathogen of concern especially strains with plasmid-mediated carbapenemase genes. The IncX3-*bla*_NDM-5_ plasmid has been described as the primary vector for *bla*_NDM-5_ dissemination. However, whether strains with this plasmid have any competitive edge remain largely unexplored. We characterized a *bla*_NDM-5_-producing *Klebsiella michiganensis* strain (KO_408) from Japan and sought to understand the driving force behind the recent dissemination of IncX3-*bl*a_NDM-5_ plasmids in different bacterial hosts. Antibiotic susceptibility testing, conjugation, and whole-genome sequencing were performed for KO_408, a clinical isolate recovered from a respiratory culture. Fitness, stability, and competitive assays were performed using the IncX3-*bla*_NDM-5_ plasmid, pKO_4-NDM-5. KO_408 was ascribed to a novel sequence type, ST256, and harbored resistance genes conforming to its MDR phenotype. The *bla*_NDM-5_ gene was localized on the ~44.9 kb IncX3 plasmid (pKO_4-NDM-5), which was transferable in the conjugal assay. The acquisition of pKO_4-NDM-5 did not impose any fitness burden and showed high stability in the host cells. However, transformants with pKO_4-NDM-5 were outcompeted by their host cells and transconjugants with the IncX3-*bla*_OXA-181_ plasmid. The genetic environment of *bla*_NDM-5_ in pKO_4-NDM-5 has been previously described. pKO_4-NDM-5 showed a close phylogenetic distance with seven similar plasmids from China. KO_408 clustered with strains within the KoI phylogroup, which is closely associated with carbapenemase genes. This study highlights the emergence of a high-risk *Klebsiella michiganensis* clone harboring carbapenemase genes and affirms that the recent spread of IncX3-*bla*_NDM-5_ plasmids might be due to their low fitness cost and stability but not their competitive prowess.

## Introduction

The drastic increase in the incidence of antibiotic-resistant strains is not only a future threat but also a present-day economic concern, especially when its prevalence among nosocomial pathogens remains soaring (Chandy et al., 2014; Hormozi et al., 2018). In particular, *Klebsiella michiganensis* is emerging as an important human pathogen that causes outbreak infections despite earlier risk being misidentified (Chapman et al., 2020; Gómez et al., 2021). This mischaracterization has downplayed this clinically relevant species in the literature (Shibu et al., 2021). *K. michiganensis* is one of nine species of the *K. oxytoca* complex within the genus *Klebsiella* (Yang et al., 2022). Members within this complex inherently produce the β-lactamase gene (*bla*_OXY_), which has evolved to help establish species-specific major phylogroups (Shibu et al., 2021). For example, *K. michiganensis* and *Klebsiella oxytoca*, which are prominent members of the complex, are affiliated with OXY-1 and OXY-2 phylogroups, respectively (Shibu et al., 2021). This chromosomal *bla*_OXY_ gene confers resistance to amino and carboxy-penicillin in *K. michiganensis* and in a similar fashion to that in other Enterobacterales, this species can also acquire extended-spectrum β-lactamases (ESBLs) and carbapenemases through horizontal transfer (Campos-Madueno et al., 2021).

Carbapenemase-producing organisms largely contribute to the extensive spread of non-susceptibility to carbapenems, exceptional broad-spectrum β-lactam antibiotics used to treat serious infections caused by ESBLs producers. Carbapanemases are classified into Ambler molecular classes A, B, and D, with New Delhi metallo-β-lactamase (NDM) as a class B enzyme (Khan et al., 2017). NDM-1 was first discovered in a clinical setting in 2008, after which 31 unique variants have been described (Feng et al., 2021). NDM-5 differs from NDM-1 by two amino substitutions and has enhanced carbapenemase activity (Hornsey et al., 2011). It was first reported in the United Kingdom from an *E. coli* strain and subsequently in other countries, including Algeria, Australia, China, India, and Japan, from different bacterial hosts. (Yaici et al., 2016; Zhu et al., 2020). *bla*_NDM-5_ has been identified on different plasmid types but is frequently located on the IncX3 plasmid, and this has been described as the primary mechanism of plasmid-mediated transfer of the *bla*_NDM-5_ gene (Flerlage et al., 2020).

Zhu and colleagues (Zhu et al., 2020) reported stability of the IncX3-*bl*a_NDM-5_ plasmid in an antibiotic-free medium, where its presence is significantly influenced by conjugal transfer. However, whether harboring IncX3-*bl*a_NDM-5_ offers any competitive advantage and if it is possibly widespread like the IncX3-*bla*_OXA-181_ plasmid remains unstudied. Here, we characterized a *bla*_NDM-5_-producing *K. michiganensis* strain (KO_408) recovered from an inpatient at a university hospital in Japan and sought to understand the driving force behind the recent dissemination of IncX3-*bla*_NDM-5_ plasmids in different bacterial hosts.

## Materials and Methods

### Patient Characteristics, Bacterial Identification, and Recombinant Strains

A 71-year-old Japanese inpatient with no history of travel outside Japan was diagnosed with pneumonia in 2018 at a university hospital in Japan. *K. oxytoca* (KO_408) was initially identified from his respiratory culture as the causative agent by matrix-assisted laser desorption/ionization time-of-flight (MALDI-TOF) mass spectrometry (Bruker Daltonics GmbH, Bremen, Germany).

Tf_Top10_-NDM-5 and Tf_C600_-NDM-5 were transformants of *Escherichia coli* Top10 and *E. coli* C600 with pKO_4-NDM-5, a plasmid derived from KO_408.

### Antimicrobial Susceptibility Testing (AST) and Characterization of Carbapenemase-Producing Organisms

The antimicrobial susceptibilities of KO_408 and the transconjugant (Tc-NDM-5) were evaluated by broth microdilution with 15 antibiotics (cefazolin, cefotaxime, piperacillin, ceftazidime, cefpodoxime, cefepime, aztreonam, gentamicin, amikacin, minocycline, imipenem, fosfomycin, levofloxacin, sulfamethoxazole/trimethoprim, and meropenem) on DP31 dry plates (Eiken Chemical Co., Tokyo, Japan). The results of minimum inhibitory concentration (MIC) values were interpreted according to guidelines outlined in the Clinical Laboratory and Standards Institute document, M100 (30th edition). Quality control was performed using *E. coli* ATCC 25922. Modified carbapenem inactivation method (mCIM) testing was performed for KO_408, as previously described (Pierce et al., 2017). The major carbapenemase genes, including those encoding VIM-, IMP-, NDM-, KPC, and OXA-48-like carbapenemases, were screened (Dallenne et al., 2010; Ayibieke et al., 2018).

### Conjugation and S1-Nuclease Pulse-Field Gel Electrophoresis Analysis

The transferability of the *bla*_NDM-5_ gene was determined by the agar mating conjugal method using a previously described protocol with some modifications (Prah et al., 2021). The sodium azide-resistant *E. coli* strain J53 was used as the recipient strain. The recipient and donor strains (KO_408) were mixed in a ratio of 1:1 and inoculated on tryptone soya agar plates containing 0.05 μg/ml meropenem. The plates were incubated at 37°C overnight, and transconjugants (Tc-NDM-5) were selected on bromothymol blue lactose agar plates containing 2 μg/ml meropenem and 100 μg/ml sodium azide. The recipients were selected using only 100 μg/ml of sodium azide. The presence of the *bla*_NDM-5_-containing plasmid was verified using PCR.

To determine the location of *bla*_NDM-5_ in KO_408 and the size of the mobile element containing the carbapenemase gene, S1-nuclease pulsed-field gel electrophoresis and Southern blotting were performed. The protocol by Prah and colleagues (Prah et al., 2021) was followed with some modifications. Genomic DNA from KO_408, Tc-NDM-5(transconjugant), Tf_Top10_-NDM-5 (transformant), and *E. coli* J53 were prepared in agarose plugs and digested with S1 nuclease (Takara Bio). DNA separation was performed on a CHEF-mapper XA system (Bio-Rad, Hercules, CA, USA) with a running time of 18 h, temperature of 14°C, field strength of 6 V/cm^2^, angles of 120°C, initial switching time of 2.2 s, and final pulse time of 63.8 s. A lambda DNA ladder (Lonza, Rockland, ME, USA) was used as the size marker. Southern blot hybridization with digoxigenin-labeled *bla*_NDM-5_ was used to determine the plasmid-carrying *bla*_NDM-5_, in accordance with the manufacturer’s instructions for the DIGHigh Prime DNA Labeling and Detection Starter Kit II (Roche Diagnostics, Germany).

### Genomic DNA Extraction, Sequencing, and Bioinformatics

DNA for genomic sequencing of KO_408 was extracted using a NucleoBond HMW DNA Kit (Takara Bio, Shiga, Japan). DNA with low molecular weights (< 40 kb) was removed using a short-read eliminator (Circulomic, Japan) prior to nanopore sequencing library preparation. It met the quantity and quality requirements for both the Oxford Nanopore and Illumina library preparations.

A nanopore sequencing library was prepared using the native barcoding expansion 1–12 kit (EXPNBD104) and the SQK-LSK109 ligation sequencing kit. Sequencing was performed for 10 h using the MinION flow cell FLO-MIN106 R9.41 in a GridION X5 sequencer. The Illumina Nextera DNA Flex Library Prep kit was used to prepare the library for Illumina short reads, and sequencing was performed using the Illumina MiSeq (San Diego, CA, USA).

Low-quality reads (MinION Q < 10; MiSeq <30) and short reads (MinION length < 500 bp; MiSeq <10 bp) were filtered out. Read yields from Illumina MiSeq and Nanopore MinION and *de novo* assembly statistics are presented in Supplementary Tables S1 and S2, respectively. A hybrid *de novo* assembly was conducted using the Unicycler v0.4.8. and genes were predicted using RAST (Overbeek et al., 2014). Antimicrobial resistance gene, multilocus sequence type (ST), and plasmid replicon type analyses were performed using ResFinder, MLST, and PlasmidFinder tools available at the Center for Genomic Epidemiology server (https://cge.cbs.dtu.dk). Isfinder (https://www-is.biotoul.fr/) and Virulence Finder Database (https://http://www.mgc.ac.cn/cgi-bin/VFs/v5/main.cgi) were used to detect mobile elements and virulence factors, respectively.

### Comparative Genomic Analysis and Phylogeny

To confirm the identity of KO_408 and delineate its lineage within the global context, average nucleotide identity (ANI) analysis of KO_408 with respect to NCBI reference genomes CP069911 (*K. oxytoca*) and CP022348(*K. michiganensis*) was initially assessed using FastANI (Jain et al., 2018). A cutoff of >95% ANI score was used as the standard for species demarcation (Jain et al., 2018). Next, all 38 completely assembled genomes of *K. michiganensis* available in the NCBI database as of February 1, 2022 were retrieved, and a further ANI analysis using CP022348 as a reference was conducted. Genomes of *K. michiganensis* with ANI values >95% and KO_408 (metadata on these genomes are presented in Supplementary Material) were annotated using Prokka (Seemann, 2014). Orthologous groups were built using a Roary pipeline (Page et al., 2015). The resultant core-genome alignment file from Roary was inputted into Iqtree to construct a phylogenetic tree using 1,000 bootstrapping replicates (Nguyen et al., 2015). The iTOL was used to visualize and annotate the trees (Letunic and Bork, 2019).

The phylogeny of the IncX3-*bla*_NDM-5_ plasmid (pKO_4-NDM-5) and 17 other plasmids sharing high homology with pKO_4-NDM-5 in a BLASTn analysis was assessed using MEGA X (Kumar et al., 2018). BRIG was used to compare pKO_4-NDM-5 with MH78170 (Alikhan et al., 2011). The genetic environment of *bla*_NDM-5_ on these plasmids and that of LC000627 was compared using EasyFig v2.1 (Sullivan et al., 2011).

### Plasmid Stability

A single colony of Tf_Top10_-NDM-5 was passaged on tryptic soy agar plate (TSA) containing no antibiotics and incubated at 37°C for 12 h. Successive passaging (200 passages) at the same interval and conditions was maintained for 100 consecutive days. The presence of the *bla*_NDM-5_-containing plasmid was analyzed after every tenth passage for selected colonies.

### Growth Kinetics

Overnight cultures of recipient *E. coli* C600, transconjugant TcEC187 (an *E. coli* C600 transconjugant containing IncX3 *bla*_OXA-181_ plasmid) described in our previous study (Prah et al., 2021), and transformant Tf_C600_-NDM-5 from TSA plates were diluted to McFarland 2 with PBS. Portions of the bacterial suspensions (200 μl) were transferred into 10 ml of sterile LB media in 18 mm diameter Pyrex tubes. The tubes were incubated at 37°C for 24 h in a Bio Shaker BR-21FP (TAITEC Co., Ltd., Japan) set to a speed of 200 rpm. The tubes were connected to a Taitec ODboxC (TAITEC Co. Ltd., Japan) for continuous measurements of the bacterial optical density (OD) at 600 nm every 30 min. Growth kinetic assays were performed in triplicate, and the mean OD was plotted against time to construct the growth curve.

### Competitive Assay

Overnight cultures of *E. coli* C600, Tf_C600_-NDM-5, and Tc1EC187 were diluted to McFarland 2 with sterile PBS. A 1:1 ratio of *E. coli* C600 and Tf-NDM-5, *E. coli* C600 and Tc1EC187, or Tc1EC187 and Tf-NDM-5 was prepared, and 30 μl was transferred into 3 ml of LB. The cultures were incubated at different time intervals (0, 6, 12, and 24 h) at 37°C with shaking (200 rpm). Cultures were serially diluted at the end of the incubation period, and 100 μl (10^−5^ diluted culture) was spread on LB agar plates with or without antibiotics. *E. coli* C600 and Tf-NDM-5 culture combinations and Tc1EC187 and Tf-NDM-5 were selected on LB agar plates with or without 2 μg/ml meropenem, whereas *E. coli* C600 and Tc1EC187 combinations were selected on LB agar plates with or without 8 μg/ml ampicillin. The plates were incubated at 37°C for 18–24 h, and the number of colonies counted. The experiment was performed in duplicate and the mean colony count was estimated.

## Results

### AST and Conjugal Transfer of *bla*_NDM-5_

KO_408 was highly resistant to all β-lactam classes of antibiotics, except the monobactam, aztreonam. Resistance to other class of antibiotics, including tetracycline and quinoline, was observed (Table 1). This clinical strain from Japan (KO_408) was mCIM-positive and harbored *bla*_NDM-5_. From the conjugal analysis, KO_408 successfully transferred *bla*_NDM-5_ to *E. coli* J53, a recipient strain. S1-PFGE and subsequent Southern hybridization with DIG-labeled *bla*_NDM-5_ showed that *bla*_NDM-5_ was localized on a mobile genetic element ∼44.9 kb in size (Supplementary Figure S1). The transconjugant strain Tc-NDM-5 also showed high resistance to most of the β-lactam antibiotics, except for aztreonam (Table 1). Tc-NDM-5 did not confer resistance to any of the antibiotics in the other classes.

**Table 1:** Minimum inhibitory concentration (MIC) profile of KO_408, Tc-NDM-5, and *E. coli* J53 in μg/mL.

Strains	β-lactam									Aminoglycoside		Tetracycline	Quinolones	others	
	PIPC	CEZ	CTX	CAZ	CFPM	CPDX	AZT	IPM	MEM	GEN	AMK	MINO	LVX	ST	FOM
KO_408	>64	>16	>32	>16	>16	>4	≤ 0.5	>8	>8	8	8	>8	>4	>32/2	<32
Tc-NDM-5	>64	>16	>32	>16	16	>4	≤ 0.5	4	8	<0.25	2	2	≤0.25	≤ 9.5/0.5	<32
*E. coli* J53	4	2	≤ 0.5	≤ 0.5	≤ 0.5	≤ 1	≤ 0.5	≤ 0.25	≤ 0.25	0.5	<1	2	≤ 0.25	≤ 9.5/0.5	<32

*PIPC, piperacillin; CEZ, cefazolin; CAZ, ceftazidime; CTX, cefotaxime; CFPM, cefepime; CPDX, cefpodoxime; AZT, aztreonam; IPM, imipenem; MEM, meropenem; FOM, Fosfomycin; MINO, GEN, gentamicin; minocycline; LVX, levofloxacin; ST, sulfamethoxazole trimethoprim; and AMK, amikacin. Changes in MIC values in E. coli J53 transconjugant (Tc-NDM-5) relative to E. coli J53 are highlighted in bold*.

### Genomic Characterization of KO_408 and Phylogeny Analysis

The ANI analysis identified KO_408 as *K. michiganensis* with an ANI score of 99.19%. Its genome comprised a chromosome of 6,018,476 bp and six other circular plasmids ranging in size from 2,569 bp to 286,463 bp (Table 2). This comprised a total of 6,037 protein-coding sequences (CDS) and accounted for a coding ratio of 87.3% (Supplementary Table S1). Related to its multidrug-resistant phenotype, KO_408 harbored resistance genes to β-lactams (*bla*_NDM-5_, *bla*_OXA-1_, and *bla*_OXY-1-7_), tetracycline (*tet(A)*), quinolone (aac(6′)-Ib-cr, *qnrS1*), and sulfonamide/dihydrofolate reductase inhibitors (*sul1*, *sul1*, *sul2*, *sul3*, and *dfrA12*) (Table 2). Most antibiotic resistance genes were plasmid-mediated, with the majority occurring in the IncHI2 plasmid (Table 2). The *bla*_NDM-5_ gene was localized to the 44,878 bp IncX3 plasmid. The chromosomally encoded β-lactamase gene variant *bla*_OXY-1-7_ was the only antibiotic resistance gene present on the chromosome (Table 2).

**Table 2:** Chromosome and plasmid features of KO_408 strain.

Strain Name	Chromosome/Plasmid	Size (bp)	MLST	Plasmid incompatibility group	pMLST	Antibiotics resistance genes
KO_408	Chromosome	6,018,476	ST265			*bla*_OXY-1-7_
	pKO_1	286,463		IncHI2	ST2	*ARR-3*, *aac(3)-Iva*, *aac(6′)-Ib-cr*, *aac(6′)-Ib-cr*, *aadA1*, *aadA2*, *aadA2*, *aph(3′)-Ia*, *aph(4)-Ia*, *bla*_OXA-1_ *catB4*, *cmlA1*, *dfrA12*, *floR*, *sul1*, *sul1*, *sul2*, *sul3*
	pKO_2	62,120		-		*qnrS1*
	pKO_3	53,503		IncR		*mph(A)*, *tet(A)*
	pKO_4-NDM-5	44,878		IncX3		*bla*_NDM-5_
	pKO_5	9,564		ColE10		
	pKO_6	2,569		-		

**Figure 1:**
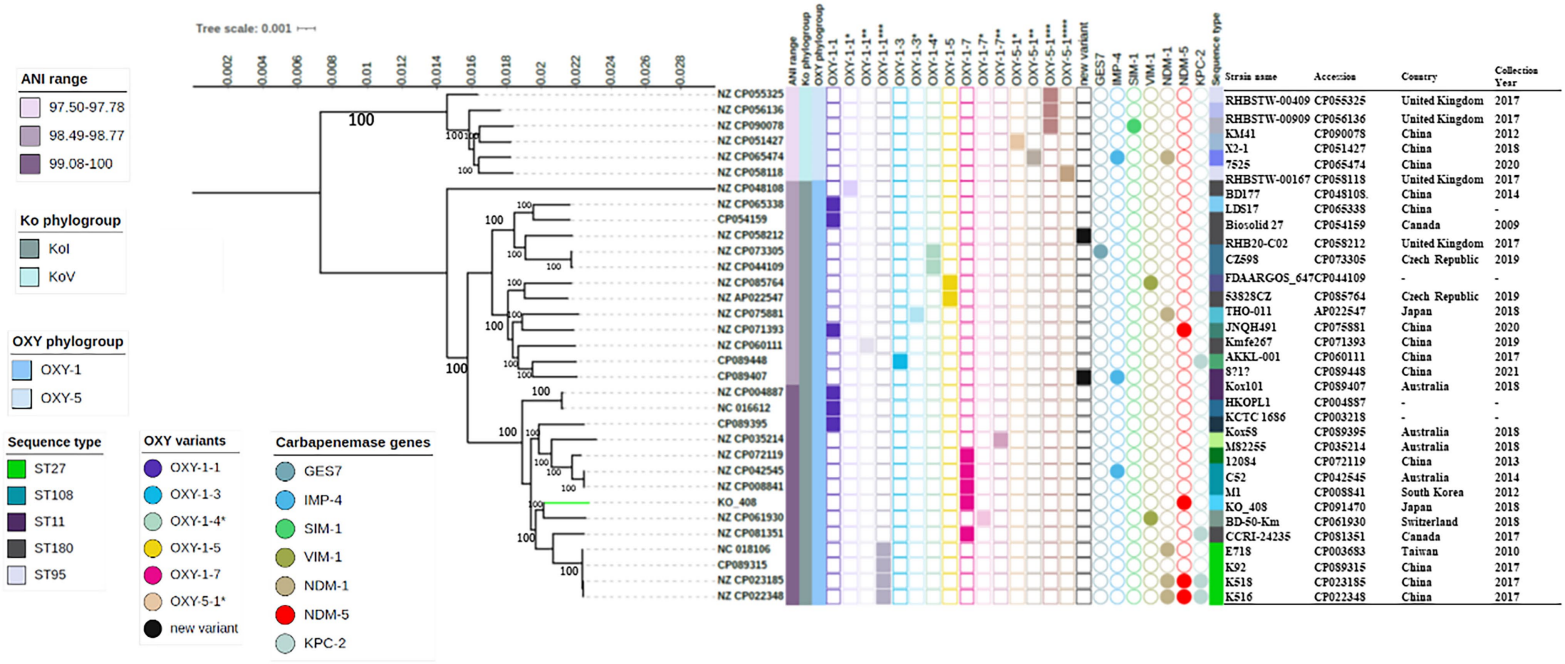
Maximum-likelihood phylogenetic tree based on *Klebsiella michiganensis* core-genomes. The tree was rooted on the node of KoV. Bootstrap values are shown next to major nodes. Strain labels are given as the strain GenBank ID, except that of KO_408. The phylogeny of KO_408 with respect to the other *K. michiganensis* genomes is highlighted with a green branch color. The major phylogroups and the OXY variants in these genomes are indicated along with their sequence types. OXY variants with an identity <99% to Resfinder references are represented on the tree as new variants, whereas those with an identity >99% but not 100% identity to the reference are shown by the query result with the * numbers representing their similarity to the reference. Sequency types (STs) with two or more frequencies are shown in the figure legend and the remaining STs are described in Supplemental_data_file1. The occurrence of carbapenemase genes in these genomes is also illustrated, as well as the genome metadata.

KO_408 was ascribed to the novel sequence type ST256. To investigate the phylogeny of KO_408 within the purview of global *K. michiganensis* collections, the resultant core-genome-based phylogenetic tree was resolved into two main groups, the KoI phylogroup with distinct subclades and the KoV phylogroup (Figure 1). The ANI range within these phylogroups relative to CP022348 was 97.50–97.78, KoV and 98.49–100, KoI (Figure 1). These ANI and phylogenetic analyses confirmed the intraspecies relatedness of the strains within these two phylogroups. Strains within the KoV phylogroup were mostly characterized by a new *bla*_OXY_ variant with >99% identity to *bla*_OXY-5-1_ (Figure 1). The KoI phylogroup was split into three sub-lineages, which did not show any specificity with the occurrence of a particular OXY variant. The occurrence of the major carbapenemase gene was not limited to any of the phylogroups, with *bla*_NDM-1_, bla_KPC-2_, and *bla*_NDM-5_ as the most frequently detected carbapenemase genes. KO_408 clustered with a subclade of KoI, which was closely associated with carbapenemase genes. This subclade predominantly contained strains with OXY-1-7 and a new OXY variant with >99% identity to *bla*_OXY-1-1_(Figure 1).

Virulence factors of KO_408 and the other strains of *K. michiganensis* isolates were investigated. Genes encoding type 1 and 3 fimbriae, iron-chelating sideropores enterobactin (ent), salmochelin, and aerobactin were mostly present in all the *K. michiganensis* genomes (Supplementary Figure S2). Iron-chelating sideropore yersiniabactin and catalase (*katA*) were mostly limited to strains in the KoI phylogroup, whereas only a few strains in both KoI and KoV phylogroups harbored genes for allantoin utilization (Supplementary Figure S2). Nearly half of the strains (n = 15/33, 45.4%) including KO_408 harbored *astA* gene, a heat stable enterotoxin, whereas only a strain belonging to the KoI phylogroup harbored the bacterial toxin colibactin (Supplementary Figure S2).

**Figure 2:**
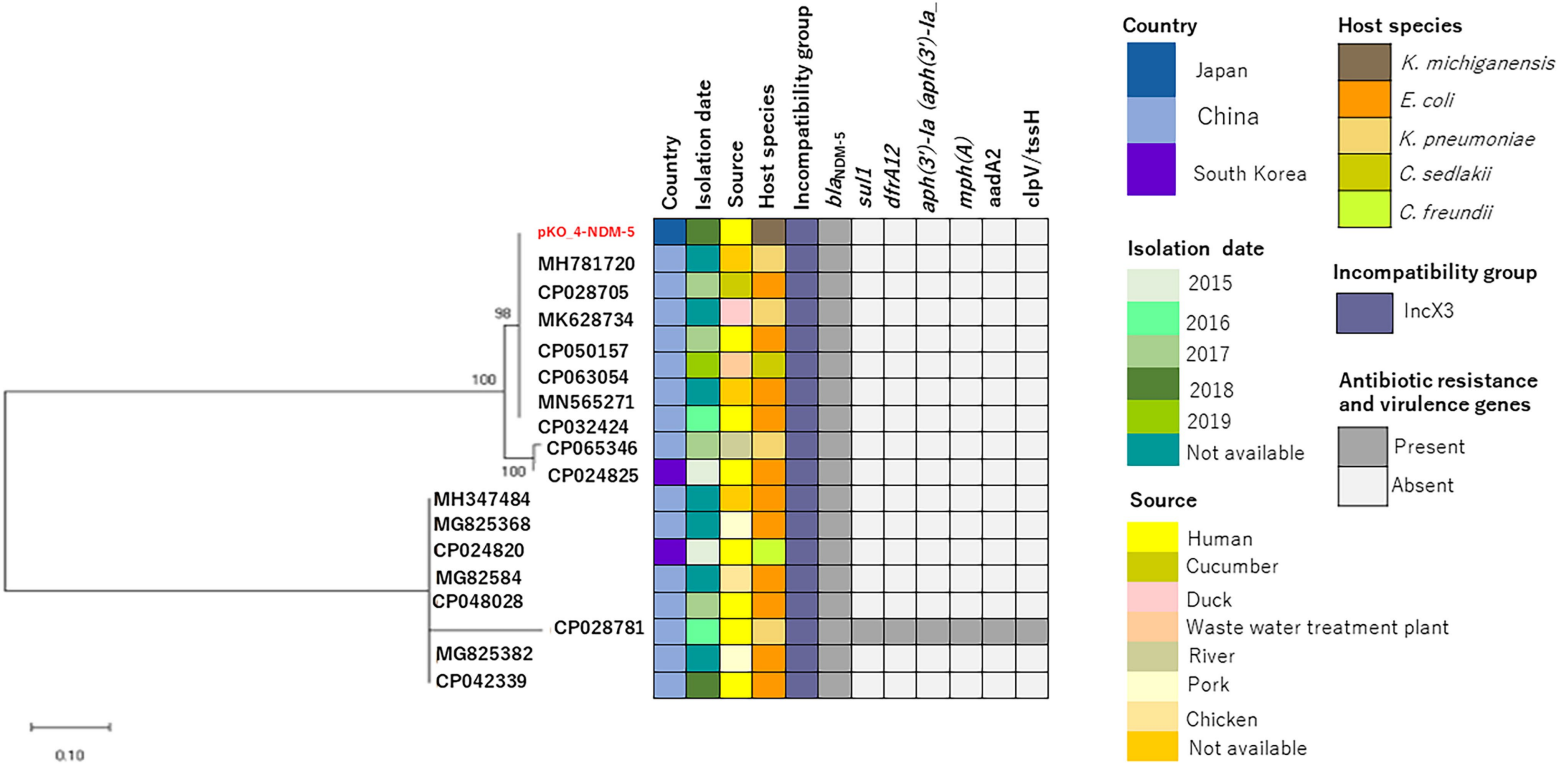
Evolutionary relatedness of pKO_4-NDM-5 and other IncX3-*bla*_NDM-5_-containing plasmids. The maximum-likelihood phylogenetic tree of these IncX3-*bla*_NDM-5_ plasmids was constructed using MEGA X software, and their bootstrap values are shown next to the branches. The plasmid metadata are also described.

**Figure 3:**
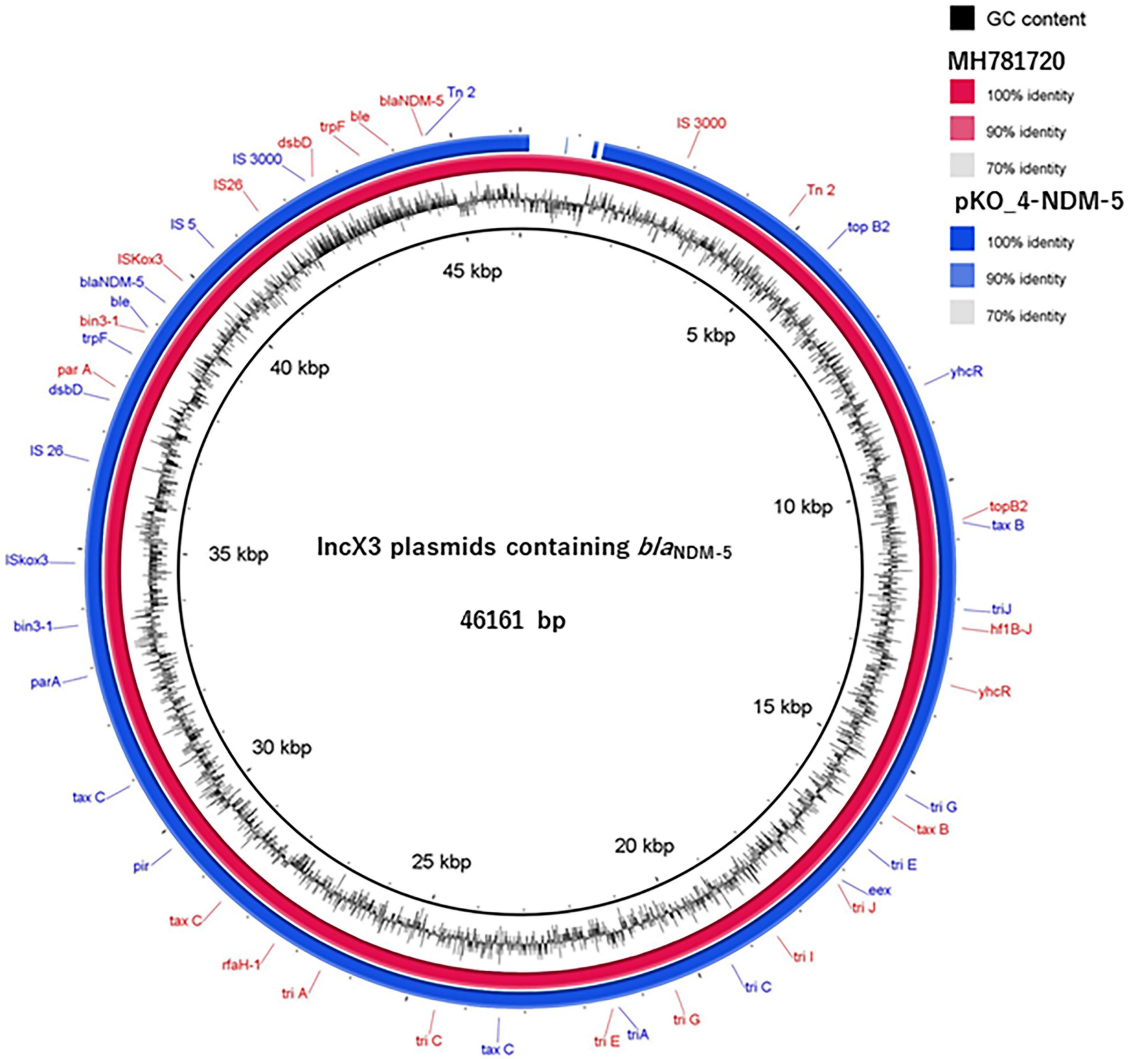
A circular comparison of pKO_4-NDM-5 and MH781720, an IncX3-*bla*_NDM-5_ containing plasmid. The homology between these two plasmids is shown by the percentage identity in the figure legend, whereas the absence of or a similarity value of <70% is indicated on the circular map as a white gap.

**Figure 4:**
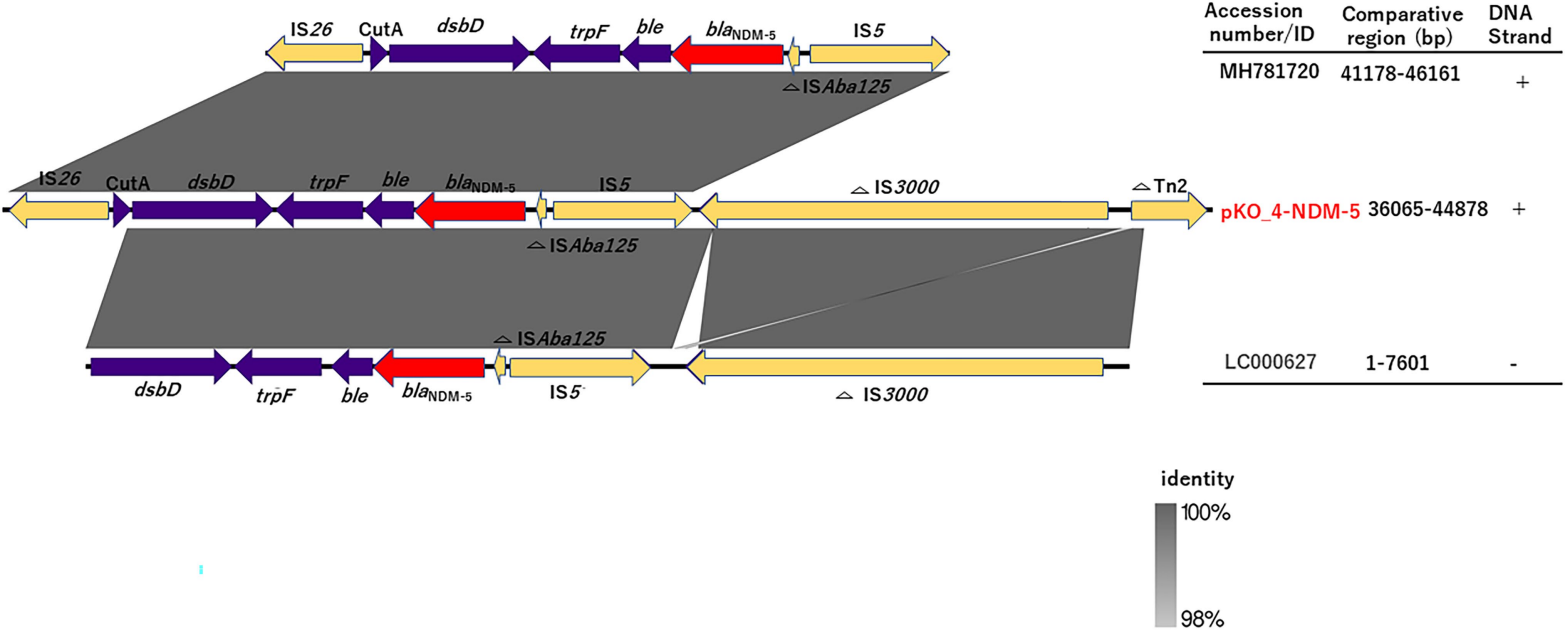
Linearized comparison of *bla*_NDM-5_ genetic environment of pKO_4-NDM-5, MH781720, and LC000627. Similar features are represented by the same color. The *bla*_NDM-5_ gene, mobile genetic elements, and other immediate CDSs are represented by red, yellow, and purple colors, respectively.

We determined the phylogenetic relatedness of the IncX3-*bla*_NDM-5_ plasmid of KO_408 (pKO_4-NDM-5) and 17 other plasmids with high homology to pKO_4-NDM-5 by BLAST analysis. pKO_4-NDM-5 shared a close genetic distance with seven plasmids from China, and these plasmids were recovered from diverse bacterial hosts and sources (Figure 2). A circular comparison of pKO-NDM-5 and MH781720 in Figure 3 shows that these IncX3 plasmids share a large, conserved scaffold containing many conjugative genes, as previously reported (Zhu et al., 2020). A structural difference that could have resulted from a deletion was observed around the genetic features closer to the *bla*_NDM-5_ portion of pKO_4-NDM-5 (Figure 3). However, a closer view of the genetic environment of *bla*_NDM-5_ in these plasmids showed previously reported genetic features. The *bla*_NDM-5_ gene was immediately followed upstream by the insertion sequence IS*Aba125* and downstream by the bleomycin resistance gene *ble*_MBL_. These were found to be bracketed within the insertion sequences IS*5* upstream and IS*26* downstream. This IS*5* and IS*26* conserved region was almost shared by the plasmid sequence of the IncN plasmid harboring the first reported case of *bla*_NDM-5_ in Japan, but the sequence lacked the insertion sequence IS26 (Figure 4).

**Figure 5:**
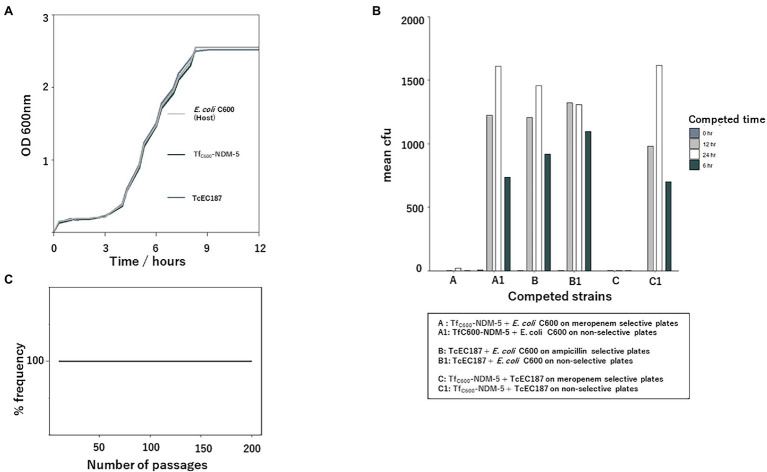
Stability, growth kinetic, and competition analyses of IncX3-*bla*_NDM-5_-containing plasmid. **(A)** Growth curve comparison of *Escherichia coli* C600, its transformant with the IncX3-*bla*_NDM-5_ plasmid, and transconjugant with the IncX3-*bla*_oxa-181_ plasmid. **(B)** Competition analysis between *E. coli* C600 with its transformant and transconjugant, as well as between the transformant and the transconjugant. **(C)** Stability analysis of IncX3-*bla*_NDM-5_-containing plasmid. The IncX3-*bla*_NDM-5_ plasmid persisted in *E. coli* Top10 after successive passaging on antibiotic-free media (12 h passage, twice daily for 100 consecutive days (200 passages) corresponding to approximately 2,400 generations of *E. coli* Top 10).

To understand the recent dissemination of IncX3-*bla*_NDM-5_-carrying plasmids in different bacterial hosts, fitness, stability, and competitive assays were performed. The growth curves of transformant bearing the IncX3-*bla*_NDM-5_ plasmid and the transconjugant bearing the IncX3-*bla*_oxa-181_-containing plasmid were compared to those of their host (*E. coli* C600). The acquisition of these IncX3-containing plasmids did not impose any fitness burden on the host cells (Figure 5). When measuring the relative fitness of strains carrying IncX3 plasmids in competing for resources in the same culture environment, Tf-NDM-5 competed with its host and TcEC187. As shown in Figure 5B, Tf-NDM-5 was outcompeted by both its host cell and TcEc187, whereas TcEC187 had similar performance with the *E. coli* host, suggesting different competitive strengths of these IncX3 plasmids. Despite the low competitiveness of strains with IncX3-*bla*_NDM5-_ plasmids, the IncX3 *bla*_NDM-5_-carrying plasmid showed strong stability in *E. coli* Top 10 in antibiotic-free culture without apparent plasmid loss for 200 consecutive passages (Figure 5).

## Discussion

The first report of the *bla*_NDM-5_ gene in Japan was from an *E. coli* isolate recovered from a traveler from Bangladesh (Nakano et al., 2014). Here, we profiled a *bla*_NDM-5_-producing *K. michiganensis* strain from an indigene in Japan and elucidated the driving factors underpinning the recent spread of the IncX3-*bla*_NDM-5_ plasmid in different bacterial host cells. The genus *Klebsiella* characteristically comprises opportunistic pathogens that cause diverse infections in humans (Saxenborn et al., 2021). However, some species in this group are under-recognized because of the ineffectiveness of conventional microbiological methods and the unavailability of reference spectra in the current clinical routine reference databases of MALDI-TOF MS to distinguish between species within this group (Saxenborn et al., 2021). KO_408 was misidentified as *K. oxytoca* by MALDI-TOF-MS; however, ANI analysis using reference genomes of *K. oxytoca* and *K. michiganensis* accurately identified KO_408 as *K. michiganensis*. This demonstrates the usefulness of ANI analysis for precisely demarcating species within *the K. oxytoca* complex.

The *K. michiganensis* clinical strain, KO_408, was multi-resistant and aside from aztreonam, it was non-susceptible to β-lactam antibiotics, including meropenem and imipenem. KO_408 harbors the *bla*_NDM-5_ gene, and these metallo-β-lactamases (MBLs) are non-hydrolyzers of monobactam such as aztreonam (Palzkill, 2013). This unique feature of MBLs highlights the clinical potential of aztreonam for the management of infections caused by MBL producers (Ramsey and MacGowan, 2016; Mauri et al., 2021).

There are limited reports on *bla*_NDM-5_-producing *K. michiganensis*. This has only been described in China from different STs (Figure 1), and the *bla*_NDM-5_ gene was found to be present on IncX3 plasmids. Its discovery in Japan indicates the gradual spread of these *bla*_NDM-5_-producing *K. michiganensis* strains, which calls for public health attention. Not all assembled genomes retrieved from the NCBI database had ANI scores above the cutoff value; thus, they were excluded from the phylogeny analysis. This affirms the incorrect assignment of some genomes within the *K. oxytoca* complex in public databases (Shibu et al., 2021). Previous findings indicate that the evolution of the *bla*_OXY_ gene parallels that of housekeeping genes within the *K. oxytoca* complex (Fevre et al., 2005; Moradigaravand et al., 2017; Shibu et al., 2021). *K. michiganensis* genomes were broadly resolved into two OXY (OXY-1 and OXY-5) and Ko (KoI and KoV) phylogroups, consistent with the current findings (Figure 1). Most OXY variants within these two phylogroups could not be fully characterized using the current Resfinder database, which limited detailed insightful analysis. K0_408 was within the OXY-1 phylogroup and in a subclade of strains with OXY-1-7 and a new OXY variant that could have evolved from *bla*_OXY-1-1_. Strains within this subclade should be on the public health radar as a potential high-risk clone for the spread of carbapenemase genes (Figure 1). The increased frequency of some virulence factors such as the siderophore yersiniabactin, genes involved in allantoin metabolism, and the cytotoxin tilivallin have been suggested to increase the severity of infections caused by species within the *K. oxytoca* complex (Cuénod et al., 2021). KO_408 did not harbor genes involved in allantoin utilization nor cytotoxins like tilivallin but had iron-chelating sideropore yersiniabactin and the heat stable enterotoxin (*astA* gene). Type 3 fimbriae which have been linked to promoting biofilm formation in *K. pneumoniae* (Schroll et al., 2010) was also detected in KO_408. Despite the occurrence of these virulence factors in KO_408, a functional study would be welcomed to establish the degree of virulence of the clinical *K. michiganensis* strain, KO_408.

Plasmid-mediated horizontal transmission of drug resistance genes is an important route for the rapid dissemination of drug resistance genes in Enterobacterales (Ma et al., 2021). *bla*_NDM-5_ was present on a conjugative IncX3 plasmid with a size of 44.9 kb. Zou and colleagues (Zou et al., 2020) compared the genetic environment of *bla*_NDM_ gene subtypes, and their analysis revealed a triad of conserved genes (*ble*_MBL_, *trpF*, and tat) downstream of *bla*_NDM_. These genes are believed to aid in conferring resistance, whereas upstream genes have varied genetic features. The *bla*_NDM-5_ gene was immediately followed by the triad genes downstream and truncated IS*Aba125* upstream. These genetic features were bracketed within IS*26* and IS*5* (Figure 4), and this finding is consistent with those of other studies (Tian et al., 2020; Zou et al., 2020; Zheng et al., 2021). Although both pKO_4-NDM-5 and MH781720 harbored IS*3000*, their relative positions were different and were thus excluded from the MH781720 genetic environment (Figures 3, 4).

The IncX3 plasmid comprises a group of plasmids with a narrow host range (Ma et al., 2021). IncX3-*bla*_NDM-5_ plasmids were first described in an *E. coli* host and subsequently in other hosts, including *Klebsiella pneumoniae*, *Citrobacter sedlakii*, and *Citrobacter freundii* (Yaici et al., 2016; Zhu et al., 2020). The analysis in Figure 2, wherein this plasmid was also found in *K. michiganensis*, indicates that these IncX3-*bla*_NDM-5_ plasmids have expanded their host preference. Thus, there is a need to understand the driving forces behind this spread.

The persistence of a plasmid in a population is dependent on several factors, including its stable maintenance and effect on host fitness (Wein et al., 2019). In the absence of positive selection for plasmid-encoded factors, the IncX3-*bla*_NDM-5_ plasmid was stably maintained by the plasmid-bearing *E. coli* Top 10 transformant cells. This suggests that antibiotic use alone is not the only factor that drives the maintenance of plasmids harboring antibiotic genes (Zhu et al., 2020). One major limitation to the spread and persistence of plasmids in bacterial populations is the fitness cost owing to the acquisition of a plasmid (Rodríguez-Beltrán et al., 2022). The acquisition of IncX3-*bla*_NDM-5_ and IncX3-*bla*_oxa-181_ plasmids by *E. coli* C600 did not result in any significant metabolic burden on the host, thus contributing to their persistence and subsequent dissemination. In the absence of selection for plasmid-related traits, plasmid-free cells are expected to outcompete plasmid-carrying cells owing to the associated fitness costs (Wein et al., 2019). Although there was no apparent difference in the fitness burden of IncX3-*bla*_NDM-5_-bearing cells and the *E. coli* C600 host cell, *E. coli* C600 outperformed the transformant cells. This outcome was consistent with the competition between IncX3-*bla*_NDM-5_- and IncX3-*bla*_oxa-181_-bearing cells. These differences in competitiveness between IncX3-*bla*_NDM-5_- and IncX3-*bla*_oxa-181_-bearing cells (Figure 5) could explain the spread of the epidemic IncX3-*bla*_oxa-181_ plasmid.

In conclusion, this study is the first to describe the isolation of a *bla*_NDM-5_-producing *K. michiganensis* strain in Japan. The strain belongs to a subclade of *K. michiganensis* strains emerging as a high-risk clone for disseminating carbapenemase genes. The findings of this plasmid study affirm that the recent dissemination of IncX3-*bla*_NDM-5_ plasmids in different bacterial hosts might be due to their low fitness burden and high stability and not to the competitive prowess of these plasmids.

## Data Availability Statement

The datasets presented in this study can be found in online repositories. The names of the repository/repositories and accession number(s) can be found at: National Center for Biotechnology Information (NCBI) BioProject database under accession number PRJNA800235.

## Ethics Statement

Ethical review and approval was not required for the study on human participants in accordance with the local legislation and institutional requirements. Written informed consent for participation was not required for this study in accordance with the national legislation and the institutional requirements.

## Author Contributions

RS, YN, and SY conceived the idea, designed the experiments, and supervised the study. IP performed the experiments and analyzed the data. RS and YN secured funding for the study. IP and RS wrote the original draft of the manuscript. All authors read and approved the final manuscript.

## Funding

This work was supported by the Japan Agency for Medical Research and Development (AMED, https://www.amed.go.jp) under grant number JP20wm0125007.

## Conflict of Interest

The authors declare that the research was conducted in the absence of any commercial or financial relationships that could be construed as a potential conflict of interest.

## Publisher’s Note

All claims expressed in this article are solely those of the authors and do not necessarily represent those of their affiliated organizations, or those of the publisher, the editors and the reviewers. Any product that may be evaluated in this article, or claim that may be made by its manufacturer, is not guaranteed or endorsed by the publisher.
