# Supplementary material for: Emergence of a High-Risk Klebsiella michiganensis Clone Disseminating Carbapenemase Genes
Source: Front Microbiol. 2022 May 23;13:880248. doi: 10.3389/fmicb.2022.880248 (PMC9169563; doi:10.3389/fmicb.2022.880248)
Supplement: Supplementary file 1 [file Table_1.docx]

**Table S1**: Filtered read statistics of Miseq and MinION data

| Sample | Reads | Number of sequences | Sum of Length | Minimum Length | Average length | Maximum  Length |
| --- | --- | --- | --- | --- | --- | --- |
| KO_408 | Miseq_1  Miseq_2 | 2,265,053  2,265,053 | 339,816,349  339,509,856 | 15  21 | 150  149.9 | 152  152 |
|  | MinION | 33,824 | 191,682,685 | 1000 | 5,667.1 | 139,612 |
