# Supplementary material for: Emergence of a High-Risk Klebsiella michiganensis Clone Disseminating Carbapenemase Genes
Source: Front Microbiol. 2022 May 23;13:880248. doi: 10.3389/fmicb.2022.880248 (PMC9169563; doi:10.3389/fmicb.2022.880248)
Supplement: Supplementary file 2 [file Table_2.docx]

**Table S2**: *De novo* hybrid assembly statistics of KO_408 genome

| Sample | Accession  Numbers | Chromosome/  Plasmids | Length(bp) | Circular/  linear | GC (%) | Average read depth | Assembler |
| --- | --- | --- | --- | --- | --- | --- | --- |
| KO_408 | CP091470  CP091471  CP091472  CP091473  CP091474  CP091475  CP092469 | Chromosome  pK0_1  pKO_2  pKO_3  pKO_4-NDM-5  pKO_5  pKO_6 | 6,018,476  286,463  62,120  53,503  44,878  9,564  2,569 | Circular  Circular  Circular  Circular  Circular  Circular  Circular | 55.88  49.39  52.74  51.27  46.76  44.88  48.8 | 41.9463  35.974  409.6135  403.3026  468.9242  8451.3967  103421.2 | unicycler  unicycler  unicycler  unicycler  unicycler  unicycler  unicycler |
