# Supplementary material for: Emergence of a High-Risk Klebsiella michiganensis Clone Disseminating Carbapenemase Genes
Source: Front Microbiol. 2022 May 23;13:880248. doi: 10.3389/fmicb.2022.880248 (PMC9169563; doi:10.3389/fmicb.2022.880248)
Supplement: Supplementary file 3 [file Table_3.docx]

**Table S3**: Annotation statistics of KO_408

| Name | value |
| --- | --- |
| Total sequence length (bp): | 6475004 |
| Number of sequences: | 6 |
| Longest sequences (bp): | 6018476 |
| N50 (bp): | 6018476 |
| Gap ratio (%): | 0 |
| GC content (%): | 55.5 |
| Number of CDSs: | 6037 |
| Average protein length: | 312.2 |
| Coding ratio (%): | 87.3 |
| Number of rRNAs: | 25 |
| Number of tRNAs: | 87 |
| Number of CRISPRs: | 3 |
