# Supplementary figures and images for: Emergence of a High-Risk Klebsiella michiganensis Clone Disseminating Carbapenemase Genes
Source: Front Microbiol. 2022 May 23;13:880248. doi: 10.3389/fmicb.2022.880248 (PMC9169563; doi:10.3389/fmicb.2022.880248)

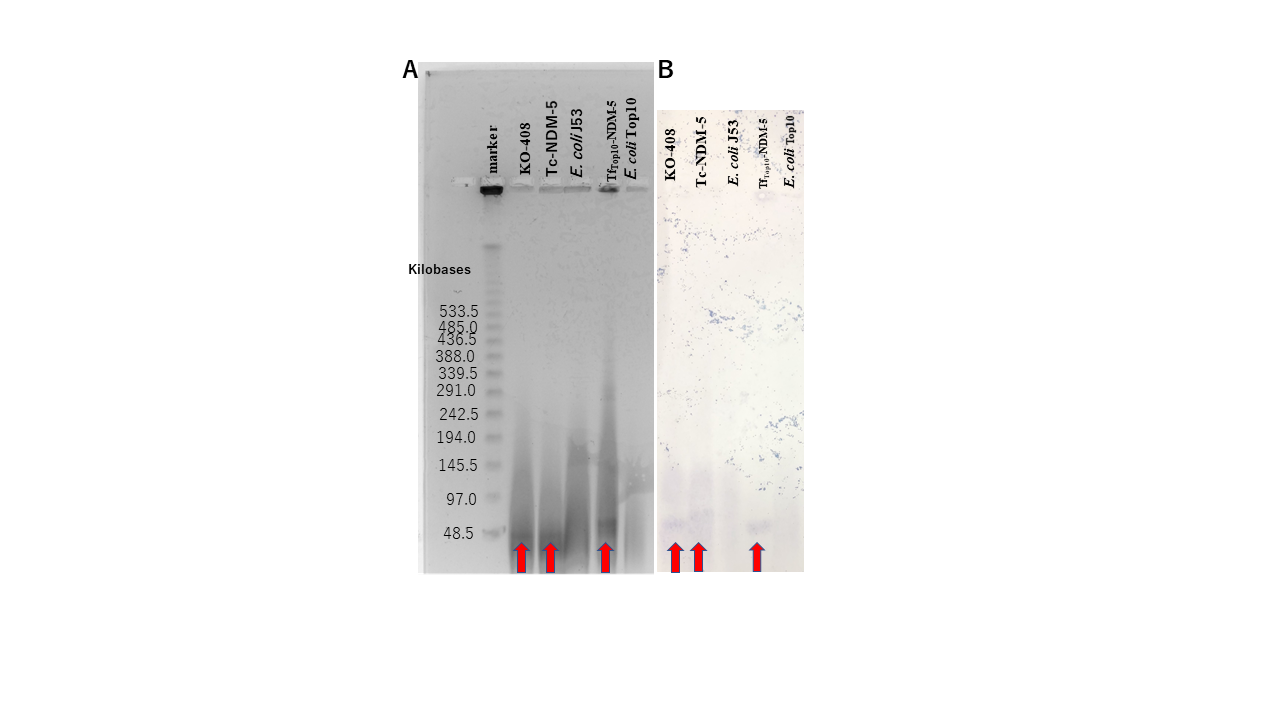

Supplement: Supplementary file 6 [file Image_1.tif]

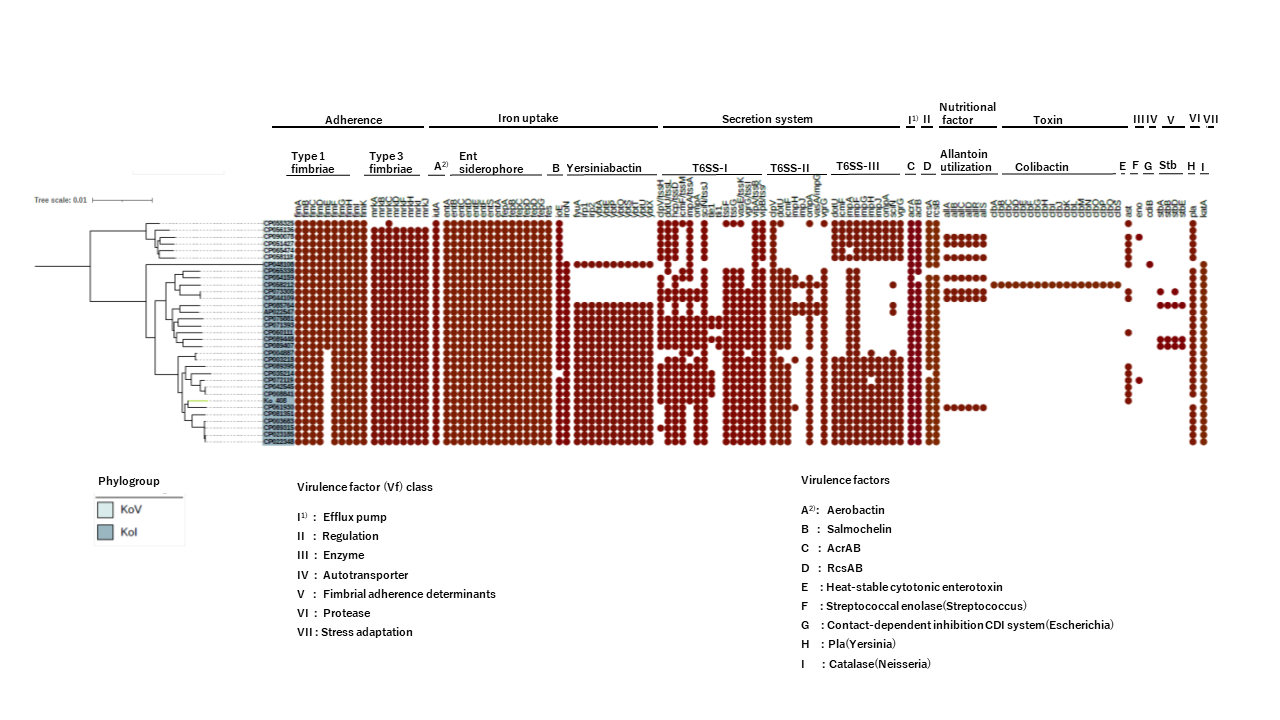

Supplement: Supplementary file 7 [file Image_2.tif]
